# Effect of Steamed Potato Bread Intake on Glucose, Lipids, and Urinary Na^+^ and K^+^: A Randomized Controlled Trial with Adolescents

**DOI:** 10.3390/ijerph17062096

**Published:** 2020-03-22

**Authors:** Haiquan Xu, Yanzhi Guo, Shijun Lu, Yunqian Ma, Xiuli Wang, Liyun Zhao, Junmao Sun

**Affiliations:** 1 Institute of Food and Nutrition Development, Ministry of Agriculture and Rural Affairs, Beijing 100081, China; guoyanzhi@caas.cn (Y.G.); lushijun@caas.cn (S.L.); mayunqian@caas.cn (Y.M.); wangxiuli01@caas.cn (X.W.); 2 National Institute for Nutrition and Health, Chinese Center for Disease Control and Prevention, Beijing 100050, China; Liyun1964@vip.sina.com

**Keywords:** potato bread, glucose, lipids, insulin, urinary Na^+^/K^+^

## Abstract

Although potatoes are highly nutritious, many epidemiological studies have connected their consumption with abnormal lipids, diabetes, and hypertension. Steamed potato bread has recently become one of China’s staple foods. A randomized controlled trial was designed to evaluate the effect of steamed potato bread consumption on Chinese adolescents. Four classes from a high school were randomly selected and assigned to the intervention group (two classes) or control group (two classes). The steamed wheat bread (100% raw wheat flour) and potato bread (raw wheat flour to cooked potato flour ratio of 3:7) were provided to the control group and intervention group as staple food once a school day for 8 weeks, respectively. Compared with the control group, the intervention group had significant net changes in systolic blood pressure (4.6 mmHg, *p* = 0.010), insulin (−4.35 mIU/L, *p* < 0.001), total cholesterol (−0.13 mmol/L, *p* = 0.032), and high-density lipoproteins cholesterol (−0.07 mmol/L, *p* = 0.010). The urinary level of Na^+^/K^+^ did not differ between the groups. In conclusion, the intake of steamed potato bread for 8 weeks resulted in positive effects on the total cholesterol and insulin profiles but a negative effect on the systolic blood pressure and high-density lipoproteins cholesterol of adolescents.

## 1. Introduction

The potato is the fourth-most consumed food in the world after rice, wheat, and corn [1,2]. Potatoes make a considerable contribution to the intake of several nutrients, such as vitamin C, potassium, and dietary fiber [3,4]. Since the national strategy of developing the potato as a staple food was implemented in 2015, potato-based food has received a great deal of interest in China [5]. Steamed bread is universally accepted as a convenient form of staple food and is crucial to most populations in China, especially in north China [6,7]. Potato flour is valuable as a replacement for wheat flour in flour production for those with cereal-based diets [8]. The addition of potato flour to wheat flour can increase the flour’s nutritional value in terms of potassium, fiber, and carotenoids. This also helps to improve the sensory quality of the bread [9,10]. Studies have obtained inconclusive findings regarding the health effects of potatoes on humans [2,4], and longstanding debates have persisted on the appropriate placement of potatoes in dietary guidance [11,12,13,14]. Nonetheless, potato is considered a healthy tuber, and the Dietary Guidelines for Chinese (2016 edition) suggest a daily potato intake of 50–100 g [15]. Some studies have revealed that potato may have negative effects on glucose metabolism because of the large amounts of rapidly absorbable starch [4]. Both epidemiologic surveys and clinical intervention trials have linked higher potato consumption to increased concentrations of fasting plasma glucose, insulin resistance, and an increased risk of type 2 diabetes mellitus [16,17,18]. However, scant research exists on how steamed potato bread made from a mixture of cooked potato flour and wheat flour affects glucose and lipids. With the hypothesis that glucose and lipids may not increase abnormally and the urinary potassium concentration could become higher after frequent intake of potato bread, this study assessed the effect of steamed potato bread intake on blood glucose, lipids, and urinary sodium (Na^+^) and potassium (K^+^).

## 2. Materials and Methods 

### 2.1. Study Design (Study Participants)

**Figure 1:**
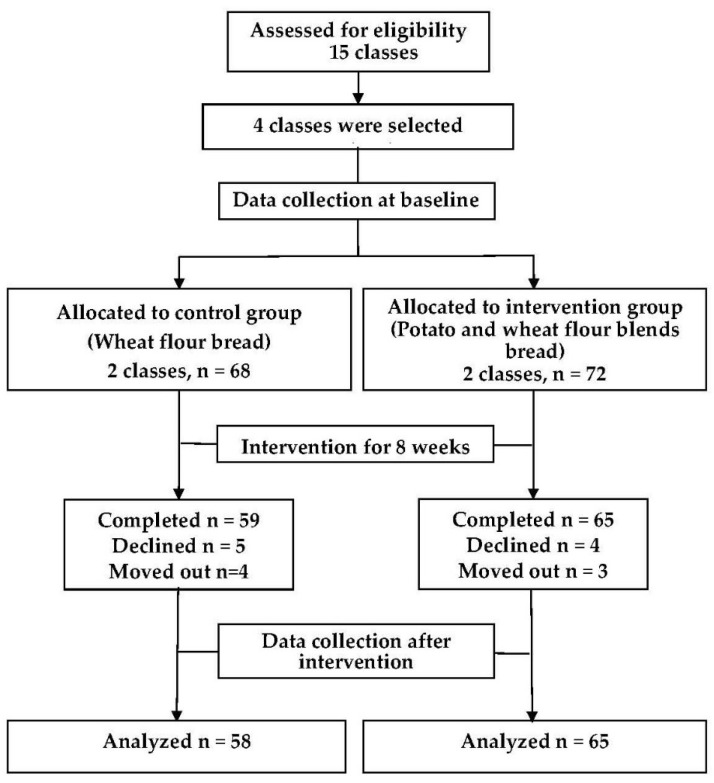
The CONSORT flowchart of the study.

This study was designed to be a randomized controlled trial. Four classes were randomly selected in the lottery in first grade from one senior middle school, and all students in the selected classes were enrolled in the trial. The four classes were randomly allocated to the intervention group (two classes) and control group (two classes) according to the random cluster method. Students from the same class participated in the same group. The intervention group was provided with steamed potato bread produced from a blend of raw wheat and cooked potato flour, and their counterparts in the control group were provided with steamed bread produced from raw wheat flour only. Dehydrated cooked potato flour was blended with wheat flour at 30% by weight to make steamed potato bread. The intervention lasted for 8 weeks. During the intervention, the steamed bread was consumed once every school day. Other foods provided to both groups were the same. Figure 1 illustrates the flow of participants through the trial. This trial was registered with the Chinese Clinical Trial Registry (ChiCTR1900027027).

Exclusion criteria for participants were as follows: (1) we excluded students with serious illnesses (such as congenital heart disease or kidney disease), those with potato or wheat flour allergies, and those who could not withstand steamed bread intake daily for 8 weeks; (2) students who recently participated in similar intervention projects were also excluded.

The trial was conducted in accordance with the Declaration of Helsinki and was approved by the China Ethics Committee of Registering Clinical Trials (Approval Number: ChiECRCT20190210). The informed consent document was voluntarily signed by participants’ parents or their guardians.

### 2.2. Assessment of Intervention Effects

Some anthropometric measurements were taken, and urine and blood indictors were evaluated both at baseline and at the end of the intervention. Fasting body weight was measured to the nearest 0.1 kg on a digital scale (RGT-140, Wujin Hengqi Co. Ltd., Changzhou, China), and height was measured using a stadiometer HP-M (Tsutsumi, Tokyo, Japan). The participants did not wear shoes and overcoats during these measurements. Body mass index (BMI) was calculated as weight in kilograms divided by height in meters squared (kg/m^2^). Blood pressure was measured by trained nurses to the nearest 2 mmHg in the seated position using a mercury sphygmomanometer with at least 10 min of rest before the measurement. The first and fifth Korotkoff sounds were used to represent systolic blood pressure (SBP) and diastolic blood pressure (DBP), respectively. Two measurements were collected for all the participants at 10 min intervals, and the average values were used for analysis.

Fasting venous blood samples (5 mL) and urine samples (10 mL) were collected in the morning after 10–14 hours of overnight fasting. Serum glucose (GLU) was determined by the glucose-oxidase method (Daiichi Pharmaceutical Co., Ltd, Tokyo, Japan) within 4 h after the sample was obtained. Total cholesterol (CHO), triglycerides (TG), low-density lipoprotein cholesterol (LDL-c), and high-density lipoprotein cholesterol (HDL-c) were determined by enzymatic methods using commercial kits (Daiichi Pharmaceutical Co., Ltd, Tokyo, Japan). Serum insulin (INS) was determined using the AxSYM based on microparticle enzyme immunoassay technology. The urine samples were collected at the first voiding after waking in the morning for the measurement of urinary sodium and potassium concentrations. Urinary Na^+^ and K^+^ concentrations (mmol/L) were measured using an ion-specific electrode method, and then the Na^+^/K^+^ molar ratio was calculated.

The analytical methods of different nutrients in bread included the Kjeldahl method (protein), Direct Drying method (water), Ignition Weight method (ash), Soxhlet Extractor method (fat), Enzymatic Gravimetric method (fiber), High-Performance Liquid Chromatography method (vitamin A, vitamin E, and β-carotene), Fluorometric method (vitamin C), inductively-coupled plasma optical emission spectrometer method (calcium, potassium, sodium and iron) and calculation method (energy and carbohydrate). The nutritional analysis indicated that the steamed potato bread provided less energy, protein, fat, carbohydrate, and vitamin E, but more fiber, vitamin B_1_, vitamin B_2_, ash, sodium, potassium, calcium, and iron than the wheat bread (Table 1). The amount of bread intake was collected with a 7-day food record.

### 2.3. Statistical Analysis

The power calculation indicated that a minimum of 55 participants for each group would be required for 80% power to detect the effect at a one-sided significance level of 0.05, according to the referred glucose parameter from an intervention study among children [19].

The average bread intake daily was calculated as the indicator of bread intake for everyone; then, the energy and nutrients provided by the steamed breads were analyzed based on the average bread intake and nutritional content of the wheat and potato bread. The continuous variables were expressed as mean and standard deviation, and binary variables were expressed as sample and percentage. The *t* test and chi-square test were used to compare differences in baseline characteristics between control and intervention groups. The mixed model was used to compare the changes of continuous variables from baseline to the end of the study between the control and intervention groups after adjustments were made for confounding factors including sex and energy intake. The statistical significance level was set at *p* < 0.05. The SAS software package version 9.2 (SAS Institute Inc, Cary, NC, USA) was used for analysis.

## 3. Results

### 3.1. General Characteristics

A total of 123 students were enrolled in the study (58 in the control group and 65 in the intervention group). The average age was 16.2 ± 0.5 years for control students and 16.4 ± 0.6 years for intervention students. The proportion of boys was statistically different between groups, at 39.0% in the control group and 69.2% in the intervention group (*p* < 0.001). Although those in the potato bread group were taller and heavier than in the wheat group at baseline, no significant difference was observed between the two groups after sex was controlled for. The intervention group consumed significantly more bread and energy from the bread than their counterparts because of the higher proportion of boys (Table 2). Compared with the control group, the intervention group got significantly more energy, protein, fat, carbohydrate, fiber, vitamin B_1_, vitamin B_2_, sodium, potassium, calcium and iron from the steamed bread daily (Table 3).

### 3.2. Physical Measurement

**Figure 2:**
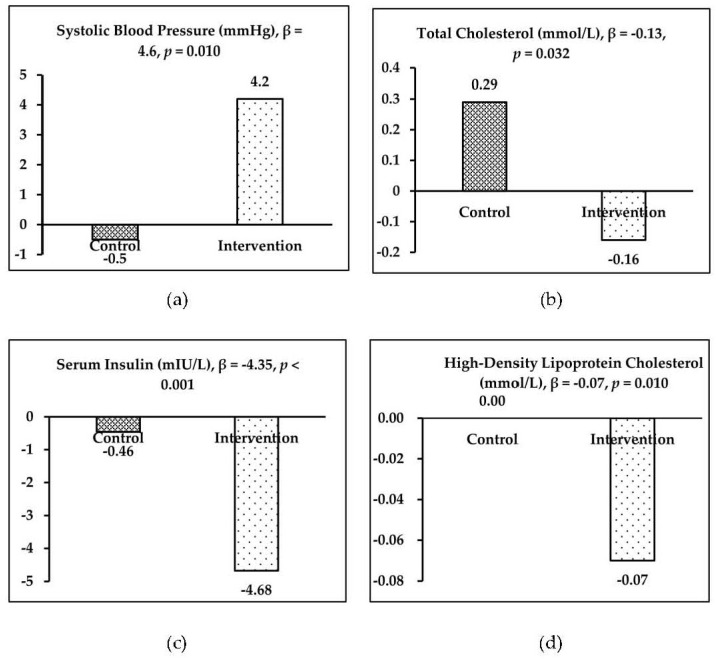
The significant intervention effects (β) in two groups: (**a**) SBP, (**b**) CHO, (**c**) INS and (**d**) HDL-c.

At baseline, the weight and height were 53.3 kg and 161.5 cm for the control group, and 60.5 kg and 168.3 cm for the intervention group. Compared with the control group, the net changes of weight and height were 0.1 kg (95% confidence interval (CI): −0.6, 0.7; *p* = 0.831) and none, respectively, for the intervention group after 8 weeks of bread intake. For blood pressure, the SBP and DBP were 119.6 mmHg and 75.6 mmHg for the control group, and 118.8 mmHg and 77.4 mmHg for the intervention group; the following net changes were observed in the intervention group: SBP increased by 4.6 mmHg (95% CI: 1.1, 8.0; *p* = 0.010) and DBP decreased by 1.3 mmHg (95% CI: −4.6, 2.0; *p* = 0.431). For the sex-based subgroup analysis, the net changes of SBP were 4.9 mmHg (95% CI: 0, 9.8; *p* = 0.049) and 3.9 mmHg (95% CI: −1.7, 9.5; *p* = 0.168) for boys and girls, respectively, and the net change of DBP was −4.8 mmHg among girls (95% CI: −9.4, −0.1; *p* = 0.044) (Table 4, Figure 2).

### 3.3. Blood Indicators

Compared with the control group, both lipid metabolic indicators (i.e., CHO, HDL-c, and LDL-c) and blood glucose metabolic indicators (i.e., GLU and INS) exhibited a decreasing trend in the intervention group after 8 weeks of intervention. Significant changes of CHO, HDL-c, and INS were observed between the groups. Compared with the control group, significant net changes (the mean difference between-group) of −0.13 mmol/L for CHO (95% CI: −0.26, −0.01; *p* = 0.032), −0.07 mmol/L for HDL−c (95% CI: −0.13, −0.02; *p* = 0.010), and −4.35 mIU/L for INS (95% CI: −5.31, −3.14; *p* < 0.001) were observed in the intervention group (Figure 2).

After the sex−based subgroup analysis, the significant net changes of CHO, HDL-c, and LDL-c were −0.28 mmol/L (95% CI: −0.43, −0.13; *p* < 0.001), −0.13 mmol/L (95% CI: −0.2, −0.05; *p* = 0.001), and −0.24 mmol/L (95% CI: −0.37, −0.11; *p* < 0.001), respectively, among boys, but no significant changes were identified in girls. Significant net changes of GLU and INS were identified in both sex subgroups: −0.31 mmol/L (95% CI: −0.49, −0.13; *p* = 0.001) and −5.95 mIU/L (95% CI: −7.39, −4.51; *p* < 0.001), respectively, for intervention boys, and 0.15 mmol/L (95% CI: −0.01, 0.32; *p* = 0.069) and 2.26 mIU/L (95% CI: −3.98, −0.55; *p* = 0.010), respectively, for intervention girls (Table 4).

### 3.4. Urinary Sodium and Potassium

Urinary Na**^+^** excretions increased by (22.5 ± 87.2) mmol/L in the intervention group (*p* = 0.057) and (26.6 ± 89.7) mmol/L in the control group (*p* = 0.043) after the intervention. No significant within-group changes for the urinary K**^+^** excretions were found. The ratio of Na**^+^**/K**^+^** increased in both groups, but no significant difference was identified between them. The same trend was found among boys and girls (Table 4).

## 4. Discussion

Although studies have indicated a potential link between potato intake and high blood glucose, abnormal lipids, and high blood pressure, the results of this trial showed that steamed potato bread intake had adverse effects on SBP but no adverse effects on glucose and DBP. The protective effect of steamed potato bread intake was also observed for CHO and INS in the intervention group; levels of these indicators did not increase. Furthermore, the results revealed that the effect of steamed potato bread intake on glucose may be different between boys and girls.

Few trials have been conducted on the effect of potato intake on human health in China, except for some epidemiological studies. To our knowledge, this is the first trial study on the effects of steamed potato bread on Chinese adolescents. Steamed potato bread is a new staple food in China made from a blend of cooked potato flour and wheat flour. Some animal model studies have revealed that potato consumption could result in reduced serum total cholesterol and lower triglycerides [4,20], which was consistent with our results. Epidemic data from the Women’s Health Study reported a positive association between potato intake and diabetes risk, but it became nonsignificant after adjustments were made for known diabetes risk factors [21]. In the Nurses’ Health Study, potato and French fry consumption were both positively associated with risk of type 2 diabetes after adjustments were made for age, dietary, and nondietary factors. However, the significant association between potato consumption and increased risk of type 2 diabetes was only found among women with a BMI of > 30 kg/m² after stratification by BMI [17]. While fried potatoes were part of a dietary pattern that was associated with an increased likelihood of type 2 diabetes in men and women [22], boiled potato was reported to be associated with a lower risk for diabetes [23,24]. Other reports have indicated an inverse relationship between potatoes and glycemia or risk of type 2 diabetes. The consumption of potatoes was inversely and independently associated with 2-hour glucose level during 20 years of follow-up [25]. The Mediterranean dietary pattern, which includes potato, has been associated with a lower predictive score for type 2 diabetes [26]. In a Japanese cohort study, potato was part of a healthy diet pattern associated with a lower risk of diabetes [4,27].

However, some cohort studies analyzing the relationship between potato consumption and human health have demonstrated negative results [28]. One large prospective cohort study revealed that higher pre-pregnancy consumption of potato was significantly associated with a higher risk of gestational diabetes mellitus, even after adjustment for risk factors such as age, family history of diabetes, physical activity, overall diet quality, and BMI [14]. Another study in women who were not pregnant found that higher consumptions of potato and French fries were associated with a moderately increased risk of type 2 diabetes mellitus after adjustment for age, dietary, and nondietary factors. In terms of potato and French fry consumption, women in the highest quintile had a 14% and 21% higher risk of type 2 diabetes than women in the lowest quintile, respectively [17]. Lea Borgi et al. concluded that a higher intake of baked, boiled, or mashed potato and French fries was independently and prospectively associated with an increased risk of developing hypertension in three large cohorts of adult men and women [29]. In our study, the SBP of adolescents in the steamed potato bread group increased by 4.6 mmHg more after 8 weeks. Additionally, whether the CHO decrease was caused by the decrease of HDL-c is worthy of study in further research.

Potato is one of the most insulinogenic foods with a high glycemic index (ranging from 71 to 106) because of the large amount of starch that is absorbed rapidly after ingestion [30,31,32]. High potato consumption could result in a sharp postprandial rise in blood glucose concentration and induce oxidative stress to pancreatic β cells, subsequently leading to β cell dysfunction or β cell exhaustion [33,34,35]. From the results of Western population studies, we are inclined to suspect that this is caused by the processing methods, but race may also play a role. Different races could have different physiological characteristics and diets after birth [36], which may lead to a different response to potato consumption. The most popular potato-based foods in Western populations are French fries and potato chips, which are high in oil, but steamed or boiled potatoes are much more common in China. One study with Chinese women revealed that the intake of tubers was associated with a lower risk of type 2 diabetes. The multivariate-adjusted relative risk of type 2 diabetes across quintiles of potato intake was 1.00, 0.82, 0.69, 0.78 and 0.72, and sweet potato intake resulted in values of 1.00, 0.54, 0.63, 0.48 and 0.51, respectively [37]. For French fries and potato chips, apart from the added oil [38], high-temperature cooking dangerously increases the acrylamide content, resulting in several harmful health effects including neurotoxicity, reproductive toxicity, carcinogenicity, genotoxicity, and mutagenicity. French fries and potato chips most likely contribute to a significant proportion of the average daily intake of acrylamide because the acrylamide precursors asparagine, glucose, and fructose are present in tubers.

Our study revealed that some beneficial effect could be found as a result of the consumption of steamed potato bread for adolescent health, which is consistent with a previous study of Chinese women. The potato flour used to produce the steamed potato bread was made from cooked potato. We suspect that the starch composition in potato bread may be different from that in uncooked potato, fried potato, and chips. The cooking time and method may have been the effective factors for glucose control. After secondary heating treatment with a cooling interval, the proportion of resistant starch in steamed potato bread might increase. Resistant starch reportedly plays a role in controlling blood glucose and insulin levels [39,40]. Additionally, potato flour contains a large amount of fiber, which is beneficial to slowing down the postprandial glucose increase [41].

Numerous studies have indicated that dietary sodium intake is associated with high blood pressure [42,43]. High consumption of potassium is associated with lower blood pressure and could counteract the negative effects of sodium on blood pressure [44,45]. The content of potassium in potato is much higher than in rice and wheat, which are other staple foods. In our study, the only dietary difference between the control and intervention groups was the intake of steamed wheat bread for the control and the intake of blended potato and wheat bread for the intervention group. The nutritional analysis indicated that the potassium content was much higher in potato bread than in wheat bread. The potassium intake of the intervention group should consequently be higher than that in the control group. We hypothesized that the urinary potassium concentration would be higher in the intervention group than in the control group, but we did not observe significant differences. Only the ratios of Na^+^/K^+^ exhibited a slight increase from baseline to the end.

This study had some potential limitations, such as the lack of detailed dietary analysis and in vitro digestibility measures, the short duration of steamed potato bread intake, and the fact that subgroups were not considered in the sample design, such as the very different proportions of boys and girls among the two groups. All these factors may have affected the results. However, this is the first clinical trial exploring the direct effect of steamed potato bread on blood glucose, lipids, and urinary Na^+^ and K^+^ in China, and it could provide valuable evaluation data for Chinese populations.

## 5. Conclusions

This 8-week trial investigating the frequent intake of steamed potato bread made from a blend of wheat flour and cooked potato flour among Chines adolescents indicated that the steamed potato bread had positive effects on the CHO and INS profiles but a negative effect on the SBP and HDL-c of adolescents, and different effects were exhibited regarding the GLU levels of boys and girls.

## Figures and Tables

**Table 1:** Nutritional content of two types of steamed bread (100 g).

Nutrients	Wheat Bread	Potato Bread
Energy (kcal)	221.1	201.0
Water (g)	45.7	49.7
Protein (g)	7.5	6.7
Fat (g)	1.1	1.0
Carbohydrate (g)	43.9	39.5
Fiber (g)	1.36	2.39
Vitamin C (mg)	<0.044	<0.044
β-Carotene (μg)	<2.00	<2.00
Vitamin E (mg)	0.387	0.266
Vitamin B_1_ (mg)	0.02	0.10
Vitamin B_2_ (mg)	0.01	0.03
Ash (g)	0.44	0.74
Sodium (mg)	74.07	81.36
Potassium (mg)	61.34	159.48
Calcium (mg)	11.88	12.27
Iron (mg)	0.60	0.71

**Table 2:** Characteristics of participants at baseline.

Characteristics	Control Group	Intervention Group
Total (*N*)	58	65
Sex (*N* (%)) ^#^		
Boys	23 (39.0)	45 (69.2) **
Girls	36 (61.0)	20 (30.8)
Nation (*N* (%)) ^#^		
Han people	54 (94.7)	61 (95.3)
Minority	3 (5.3)	3 (4.7)
Menarche/spermorrhea (*N* (%)) ^#^		
Yes	56 (94.9)	57 (87.7)
No	3 (5.1)	8 (12.3)
Age (year, Mean (SD)) ^†^	16.2 (0.5)	16.4 (0.6)
Height (cm, Mean (SD)) ^†^	161.5 (7.8)	168.3 (7.9) *
Weight (kg, Mean (SD)) ^†^	53.3 (8.2)	60.5 (11.9) *
BMI (kg/m^2^, Mean (SD))	20.4 (2.5)	21.2 (3.4)
Bread intake (g/day, Mean (SD)) ^†^	119.4 (47.3)	160.8 (70.1) *
Energy provided by bread (kcal/day, Mean (SD)) ^†^	264.0 (104.6)	323.3 (140.9) *

** *p* < 0.01. * *p* < 0.05. ^#^ Comparison by chi-square test. ^†^ Comparison by *t* test. BMI: body mass index.

**Table 3:** Nutrients provided by the steamed breads daily (Mean ± SD).

Nutrients	Control Group	Intervention Group	*p*-Value
Energy (kcal)	264.0 (104.6)	323.3 (140.9)	0.003
Protein (g)	8.9 ± 3.5	10.8 ± 4.4	0.005
Fat (g)	1.3 ± 0.5	1.6 ± 0.7	0.003
Carbohydrate (g)	52.3 ± 20.4	64.0 ± 25.7	0.004
Fiber (g)	1.62 ± 0.63	3.87 ± 1.55	<0.001
Vitamin E (mg)	0.46 ± 0.18	0.43 ± 0.17	0.317
Vitamin B_1_ (mg)	0.02 ± 0.01	0.16 ± 0.07	<0.001
Vitamin B_2_ (mg)	0.01 ± 0	0.05 ± 0.02	<0.001
Sodium (mg)	88.32 ± 34.44	131.71 ± 52.92	<0.001
Potassium (mg)	73.14 ± 28.52	258.18 ± 103.74	<0.001
Calcium (mg)	14.16 ± 5.52	19.86 ± 7.98	<0.001
Iron (mg)	0.72 ± 0.28	1.15 ± 0.46	<0.001

The *t* test was used for the comparison between groups.

**Table 4:** Outcomes of the intervention for groups and subgroups.

Subgroups	Variables	Control Group		Intervention Group		Effect	
		Baseline	Changes	Baseline	Changes	Beta (95% CI)	*p*-Value
**Overall**	Weight (kg)	53.3 ± 8.2	1.9 ± 1.4 **	60.5 ± 12.1	1.9 ± 2.0 **	0.1 (−0.6, 0.7)	0.831
	Height (cm)	161.5 ± 7.8	1.8 ± 0.8 **	168.3 ± 8.0	1.7 ± 1.1 **	0 (−0.4, 0.3)	0.858
	BMI (kg/m^2^)	20.4 ± 2.5	0.3 ± 0.6 **	21.2 ± 3.4	0.2 ± 0.8 *	0 (−0.3, 0.2)	0.738
	SBP (mmHg)	119.6 ± 9.3	−0.5 ± 0.3	118.8 ± 8.9	4.2 ± 8.8 **	4.6 (1.1, 8.0)	0.010
	DBP (mmHg)	75.6 ± 8.0	−1.9 ± 8.7	77.4 ± 9.2	−3.2 ± 9.2 **	−1.3 (−4.6, 2.0)	0.431
	GLU (mmol/L)	4.45 ± 0.3	0.53 ± 0.28 **	4.40 ± 0.37	0.46 ± 0.36 **	−0.07 (−0.19, 0.04)	0.215
	INS (mIU/L)	11.38 ± 3.1	−0.46 ± 3.00	14.31 ± 4.34	−4.68 ± 2.98 **	−4.35 (−5.31, −3.14)	<0.001
	CHO (mmol/L)	3.42 ± 0.55	0.29 ± 0.33 **	3.39 ± 0.49	0.16 ± 0.35 **	−0.13 (−0.26, −0.01)	0.032
	HDL-c (mmol/L)	1.32 ± 0.26	0.00 ± 0.16	1.24 ± 0.25	−0.07 ± 0.14 **	−0.07 (−0.13, −0.02)	0.010
	LDL-c (mmol/L)	1.70 ± 0.5	0.08 ± 0.43	1.75 ± 0.42	−0.02 ± 0.23 **	−0.10 (−0.23, 0.03)	0.132
	TG (mmol/L)	0.78 ± 0.25	0.0 ± 0.24	0.82 ± 0.28	0.06 ± 0.27	0.06 (−0.03, 0.15)	0.198
	Urinary Na^+^ (mmol/L)	150.6 ± 70.3	26.6 ± 89.7 *	187.6 ± 80.4	22.5 ± 87.2	−4.2 (−38.3, 30.0)	0.809
	Urinary K^+^ (mmol/L)	21.6 ± 17.6	1.0 ± 22.2	26.6 ± 17.5	−0.7 ± 20.8	−1.7 (−10.0, 6.6)	0.689
	Urinary NA^+^/K^+^	9.4 ± 4.4	1.5 ± 6.0	8.7 ± 3.9	1.9 ± 5.7 *	0.5 (−1.8, 2.7)	0.687
**Boys**	Weight (kg)	59.0 ± 9.4	1.4 ± 1.4 **	63.7 ± 12.8	1.8 ± 2.0 **	0.3 (−0.6, 1.3)	0.478
	Height (cm)	169.4 ± 4.8	2.0 ± 0.9 **	171.7 ± 6.7	2.0 ± 1.2 **	0 (−0.6, 0.6)	0.959
	BMI (kg/m^2^)	20.6 ± 3.2	0 ± 0.5	21.6 ± 3.9	0.1 ± 0.8	0.1 (−0.3, 0.4)	0.660
	SBP (mmHg)	120.7 ± 8.4	−0.4 ± 9.7	120.3 ± 7.3	4.5 ± 9.1 *	4.9 (0, 9.8)	0.049
	DBP (mmHg)	74.4 ± 8.6	−3.3 ± 8.9	76.8 ± 9.5	−1.9 ± 9.8	1.4 (−3.6, 6.4)	0.580
	GLU (mmol/L)	4.36 ± 0.29	0.72 ± 0.30 **	4.42 ± 0.4	0.41 ± 0.37 **	−0.31 (−0.49, −0.13)	0.001
	INS (mIU/L)	10.02 ± 1.91	0.71 ± 2.46	14.96 ± 4.89	−5.25 ± 2.88 **	−5.95 (−7.39, −4.51)	<0.001
	CHO (mmol/L)	3.10 ± 0.56	0.40 ± 0.23 **	3.29 ± 0.44	0.12 ± 0.32 *	−0.28 (−0.43, −0.13)	<0.001
	HDL-c(mmol/L)	1.23 ± 0.23	0.05 ± 0.15	1.15 ± 0.2	−0.08 ± 0.13 **	−0.13 (−0.2, −0.05)	0.001
	LDL-c(mmol/L)	1.47 ± 0.54	0.21 ± 0.29	1.70 ± 0.39	−0.03 ± 0.22 **	−0.24 (−0.37, −0.11)	<0.001
	TG (mmol/L)	0.75 ± 0.24	−0.05 ± 0.21	0.89 ± 0.28	0 ± 0.22	0.05 (−0.06, 0.17)	0.338
	Urinary Na^+^ (mmol/L)	139.8 ± 57.3	45.1 ± 78.9 *	189.6 ± 80.4	21.0 ± 86.1	−24.1 (−68.2, 19.9)	0.278
	Urinary K^+^ (mmol/L)	20.2 ± 16.2	1.9 ± 24.5	27.3 ± 18.9	−2.5 ± 19.5	−4.4 (−15.6, 6.8)	0.436
	Urinary NA^+^/K^+^	9.0 ± 4.1	1.9 ± 5.1	8.8 ± 4.0	2.1 ± 5.2 *	0.2 (−2.5, 2.9)	0.896
**Girls**	Weight (kg)	49.8 ± 4.9	2.1 ± 1.4 **	53.5 ± 6.4	2.3 ± 2.2 **	0.1 (−1, 1.3)	0.787
	Height (cm)	156.7 ± 4.8	1.6 ± 0.8 **	161.0 ± 5.2	1.3 ± 0.7 **	−0.4 (−0.8, 0.1)	0.087
	BMI (kg/m^2^)	20.3 ± 2.0	0.4 ± 0.5 **	20.6 ± 2.1	0.5 ± 0.8 **	0.1 (−0.3, 0.5)	0.663
	SBP (mmHg)	118.9 ± 9.9	−0.3 ± 10.7	115.6 ± 11.3	3.6 ± 8.5	3.9 (−1.7, 9.5)	0.168
	DBP (mmHg)	76.4 ± 7.6	−0.9 ± 8.6	78.7 ± 8.8	−5.7 ± 7.6 **	−4.8 (−9.4, −0.1)	0.044
	GLU (mmol/L)	4.50 ± 0.30	0.41 ± 0.20 **	4.36 ± 0.29	0.57 ± 0.33 **	0.15 (−0.01, 0.32)	0.069
	INS (mIU/L)	12.27 ± 3.42	−1.21 ± 3.10 *	12.84 ± 2.19	−3.47 ± 2.90 **	−2.26 (−3.98, −0.55)	0.010
	CHO (mmol/L)	3.64 ± 0.43	0.22 ± 0.36 **	3.61 ± 0.54	0.24 ± 0.41 *	0.02 (−0.2, 0.23)	0.882
	HDL-c (mmol/L)	1.38 ± 0.27	−0.03 ± 0.15	1.44 ± 0.24	−0.06 ± 0.16	−0.03 (−0.11, 0.06)	0.540
	LDL-c (mmol/L)	1.84 ± 0.42	−0.01 ± 0.48	1.87 ± 0.45	0 ± 0.26	0.01 (−0.22, 0.25)	0.909
	TG (mmol/L)	0.79 ± 0.26	0.03 ± 0.26	0.67 ± 0.23	0.17 ± 0.32*	0.15 (−0.01, 0.31)	0.071
	Urinary Na^+^ (mmol/L)	157.8 ± 77.7	11.6 ± 96.5	182.3 ± 82.7	26.7 ± 93.2	15.1 (−46.9, 77.2)	0.624
	Urinary K^+^ (mmol/L)	22.5 ± 18.6	0.3 ± 20.7	24.9 ± 13.4	4.4 ± 24.1	4.1 (−10.1, 18.4)	0.561
	Urinary NA^+^/K^+^	9.7 ± 4.6	1.1 ± 6.8	8.5 ± 3.6	1.5 ± 7.3	0.4 (−4.1, 4.9)	0.864

The linear growth model was used for the comparison between groups. The comparison of overall participants was adjusted for sex. The *t* test was used for comparison within-group; ** *p* < 0.01; * *p* < 0.05. BMI: body mass index; SBP: systolic blood pressure; DBP: diastolic blood pressure; GLU: serum glucose; INS: serum insulin; CHO: total cholesterol; HDL-c: high-density lipoprotein cholesterol; LDL-c: low-density lipoprotein cholesterol; TG: triglycerides.
